# Epigenetic signature of very low birth weight in young adult life

**DOI:** 10.1038/s41390-024-03354-6

**Published:** 2024-06-19

**Authors:** Juho Kuula, Darina Czamara, Helena Hauta-alus, Jari Lahti, Petteri Hovi, Maija E. Miettinen, Justiina Ronkainen, Johan G. Eriksson, Sture Andersson, Marjo-Riitta Järvelin, Sylvain Sebert, Katri Räikkönen, Elisabeth B. Binder, Eero Kajantie

**Affiliations:** 1 Population Health Research, Finnish Institute for Health and Welfare, Helsinki, Finland; 2 HUS Medical Imaging Center, Department of Radiology, University of Helsinki and Helsinki University Hospital, Helsinki, Finland; 3 Department of Translational Research in Psychiatry, Max-Planck-Institute of Psychiatry, Munich, Germany; 4 PEDEGO Research Unit, MRC Oulu, Oulu University Hospital and University of Oulu, Oulu, Finland; 5 Children’s Hospital, Pediatric Research Center, University of Helsinki and Helsinki University Hospital, Helsinki, Finland; 6 Research Program for Clinical and Molecular Metabolism, Faculty of Medicine, University of Helsinki, Helsinki, Finland; 7 Department of Psychology and Logopedics, University of Helsinki, Helsinki, Finland; 8 Center for Life Course Health Research, University of Oulu, Oulu, Finland; 9 Folkhälsan Research Centre, Topeliusgatan 20, 00250 Helsinki, Finland; 10 Singapore Institute for Clinical Sciences, Agency for Science, Technology and Research, Singapore, Singapore; 11 Department of Obstetrics & Gynaecology and Human Potential Translational Research Programme, Yong Loo Lin School of Medicine, National University of Singapore, Singapore, Singapore; 12 Department of Epidemiology and Biostatistics, School of Public Health, Imperial College London, London, UK; 13 Clinical Medicine Research Unit, University of Oulu, Oulu, Finland; 14 Department of Clinical and Molecular Medicine, Norwegian University of Science and Technology, Trondheim, Norway

## Abstract

**Background:**

Globally, one in ten babies is born preterm (<37 weeks), and 1–2% preterm at very low birth weight (VLBW, <1500 g). As adults, they are at increased risk for a plethora of health conditions, e.g., cardiometabolic disease, which may partly be mediated by epigenetic regulation. We compared blood DNA methylation between young adults born at VLBW and controls.

**Methods:**

157 subjects born at VLBW and 161 controls born at term, from the Helsinki Study of Very Low Birth Weight Adults, were assessed for peripheral venous blood DNA methylation levels at mean age of 22 years. Significant CpG-sites (5’—C—phosphate—G—3’) were meta-analyzed against continuous birth weight in four independent cohorts (pooled *n* = 2235) with cohort mean ages varying from 0 to 31 years.

**Results:**

In the discovery cohort, 66 CpG-sites were differentially methylated between VLBW adults and controls. Top hits were located in *HIF3A*, *EBF4,* and an intergenic region nearest to *GLI2* (distance 57,533 bp). Five CpG-sites, all in proximity to *GLI2*, were hypermethylated in VLBW and associated with lower birth weight in the meta-analysis.

**Conclusion:**

We identified differentially methylated CpG-sites suggesting an epigenetic signature of preterm birth at VLBW present in adult life.

**Impact:**

- Being born preterm at very low birth weight has major implications for later health and chronic disease risk factors.
- The mechanism linking preterm birth to later outcomes remains unknown.
- Our cohort study of 157 very low birth weight adults and 161 controls found 66 differentially methylated sites at mean age of 22 years.
- Our findings suggest an epigenetic mark of preterm birth present in adulthood, which opens up opportunities for mechanistic studies.

## Background

Globally, ~10% of all children are born preterm (<37 gestational weeks) and 1–2% very preterm (VPT, <32 weeks) or with a very low birth weight (VLBW; <1500 g).^1^ Cohort studies have shown that adults born preterm at VLBW have poorer blood glucose regulation,^2^ decreased bone mineral density,^3^ higher blood pressure,^4^ poorer lung function,^5^ poorer cognitive abilities^6,7^ and a particular “preterm behavioral phenotype” that may predispose to anxiety disorders and depression,^8^ with many of these also observable in low birth weight (LBW; <2500 g) adults. The differences in these outcomes between individuals born at VLBW range from moderate (~0.5 SD) to large (~0.8 SD) when compared with individuals born at term or with normal birth weight.^9^

Epigenetic modifications including DNA methylation (DNAm) have been suggested as mediators between early life exposures and later health outcomes.^10,11^ In utero exposure to maternal and environmental factors have been linked to epigenetic modifications in the offspring, exemplified by maternal smoking associating with epigenetic changes in newborns, children, adolescents, and middle-aged individuals.^12,13^ Prenatal maternal stress has also been linked to DNAm changes in the offspring.^14,15^

Individuals born preterm at VLBW serve as a model for studying survivors of extreme adverse early life events. Their comprehensive phenotyping and epigenotyping offer valuable insights into the mechanisms of adverse early life conditions potentially influencing long-term health.^16^ Differences in DNAm between VLBW or VPT subjects and term controls have been reported in a few studies. Epigenetic changes in VLBW subjects have most clearly been seen in the neonatal period and infancy,^17,18^ and emerging evidence suggests that some differentially methylated positions (DMPs) are present in adolescence.^19^ Only few epigenetic studies of VLBW individuals have been published beyond adolescence and they are relatively small-scale,^20^ with the largest and longest follow-up of 45 extremely low birth weight (ELBW, <1000 g) individuals and 47 controls being reported by Mathewson et al. in 32-year-olds.^21^ A recent study by Cameron et al. compared DNAm at birth (109 VLBW individuals, 52 controls) and in 28-year-olds (215 VLBW individuals, 96 controls).^22^ No robust DMPs across these studies have emerged. There is little evidence of overlap of DMPs between studies examining VLBW and LBW individuals. Meta-analyses assessing both gestational age^18^ and birth weight^19^ suggest that specific regions in the epigenome may be more likely to be linked to prematurity than others, but results remain inconclusive. As these studies have been conducted in cohorts with varying exposures, it is plausible that the DNAm differences are consequences of adverse pre- and perinatal conditions and not causes for preterm birth.

We investigated DNAm differences in adulthood in three steps. First, we compared DNAm differences in 157 young adults born at VLBW and 161 term controls. Second, we validated our findings in relation to birth weight meta-analyzing four independent birth cohorts with an overall sample-size of 2235 individuals spanning an age range of 0–31 years. Third, we extended our exploration of DNAm differences to include secondary exposures (gestational age, birth weight SD, and being born small for gestational age (SGA, <–2 birth weight SD)).

We hypothesized that there are blood DNAm differences between young adults born preterm at VLBW and term controls. We further hypothesized that some of these differences may be seen in unrelated cohorts of varying ages when investigating birth weight as the exposure. We also hypothesized that some of the differences in DNAm may be related to secondary birth exposures.

## Material and methods

### Discovery study population

Briefly, the original cohort of HeSVA (Helsinki Study of Very Low Weight Adults) consists of 335 children born at VLBW between 1978 and 1985 and treated at the neonatal intensive care unit in Helsinki University Central Hospitals Children’s Hospital, the tertiary center serving the province of Uusimaa, Finland. For the current study, 166 VLBW subjects and 172 term controls, group-matched as described previously by age, sex, and birth hospital attended a follow-up visit in 2004 at mean age of 22.5 years of age.^2,3,23^ The examination included a fasting blood sample for DNA extraction. In case of multiple pregnancy, we included one subject chosen at random from each family to dilute the possibility of familial bias or inflation, leaving 157 VLBW subjects.

### Follow-up cohorts and age groups

Follow-up and validation of the discovery cohort’s findings was performed in four independent cohorts of varying age groups: PREDO (Prediction and Prevention of Preeclampsia and Intrauterine Growth Restriction, *n* = 817)^24^ at birth, GLAKU (Glycyrrhizin in Licorice, *n* = 236)^25,26^ at mean age of 12 years, NFBC1986 (Northern Finland Birth Cohort 1986, *n* = 479)^27,28^ at mean age of 16 years and NFBC1966 (Northern Finland Birth Cohort 1966, *n* = 703)^29^ at mean age of 31 years.

### PREDO (at birth/newborn subjects)

Briefly, PREDO is a prospective multicenter study of 1079 Finnish women with known risk-factor status for pre-eclampsia and intrauterine growth restriction. The women gave birth to a singleton live child between 2006 and 2010. Fetal cord blood DNAm data were available from 963 children, and 817 samples were available for the current analysis.

### GLAKU (12–13-year-old subjects)

GLAKU is an urban-based cohort of 1049 women who gave birth to a singleton live child between March and November 1998. Between 2009-2011, 920 children were invited for a follow-up visit and 451 participated at early adolescence. Of the participating children, peripheral blood DNAm data were available from 236 children.

### NFBC1986 and NFBC1966 (16-, 31- and 46-year-old subjects)

NFBC1986 and NFBC1966 are prospective birth cohorts of individuals born in the two northernmost provinces (Oulu and Lapland) of Finland in 1966 and 1986 and followed through life. NFBC1966 comprises of 12,231 children and their parents who were originally recruited based on their expected delivery date being between January 1 and December 31, 1966. NFBC1986 comprises of 9479 children (9432 live births) whose mothers were recruited based on an expected date of birth between July 1, 1985, and June 30, 1986. In the current study, peripheral venous blood DNAm data from 510 (NFBC1986 at 16 years), 722 (NFBC1966 at 31 years) and 705 (NFBC1966 at 46 years) randomly selected individuals were available.

### Exposure variables, covariates, DNA extraction, and DNAm data

Fasting peripheral venous EDTA blood samples, anthropometric measurements, and questionnaire data regarding health and lifestyle were collected. Blood samples were stored at -20 C for later analyses. Genomic DNA was extracted from EDTA-anti-coagulated whole peripheral blood by using QIAamp DNA Blood Maxi Kit (Qiagen®).

Peripheral venous blood DNA was available from the discovery cohort’s 157 VLBW unrelated subjects and 161 term controls. Illumina Infinium HumanMethylation450 BeadChip (Illumina, San Diego, California) was used to determine genome wide DNAm levels. Pre-processing was performed using the R-package *minfi*.^30^ Raw beta-values were normalized using functional normalization, CpG-sites (5’—C—phosphate—G—3’) failing in more than 50% of the samples (based on a detection *p* value of 0.10) were removed. Batch-correction was performed using *combat*^31^ and non-autosomal CpGs as well as cross-hybridizing CpGs were removed. The final dataset included 428,759 CpG-sites and 157 VLBW cases and 161 controls. CpG-sites were mapped to genomic locations using Illumina’s annotation using the R-package *minfi*.^30^ The replication cohort’s methylation analyses are based on cord blood (PREDO, *n* = 817) and peripheral venous blood (GLAKU, *n* = 236; NFBC1986 *n* = 479; and NFBC1966, *n* = 703) and are described in detail previously.^25,32,33^ Cell-type proportions were estimated using the Houseman method for peripheral venous blood^34^ and the Bakulski algorithm for cord blood.^35^ Further details of cohort-specific methods are detailed in the Supplementary Methods.

### Statistical analyses

Analyses were conducted using VLBW/term-dichotomization as the primary exposure for the discovery cohort, and birth weight in grams as a continuous exposure variable for PREDO and GLAKU cohorts. Linear regression was applied adjusting for blood cell type proportions, age at sampling, sex, maternal smoking in pregnancy (classified as smoking/non-smoking during pregnancy) and parental socioeconomic status or highest attained parental education. Beta values are expressed as either positive or negative (VLBW = 0, control = 1, i.e., a positive beta corresponds to more methylation in controls and hypomethylation in the VLBW group). For NFBC cohorts we used the same regression models against birth weight as a continuous variable, as well as a dichotomous prematurity-status (<37 gestational weeks).

A meta-analysis of the results of PREDO, GLAKU, NFBC1986, and NFBC1966 was conducted using the R-package *metafor*,^36^ with continuous birth weight as the exposure. NFBC1966 had two time points available, 31 and 46 years of age. The time point of 31 years was used, as more samples were available and it was closer to our discovery cohort’s age. Multiple testing was controlled for using Benjamini-Hochberg false discovery rate (FDR) method. The CpG-sites from the discovery analysis with FDR-adjusted *p* < 0.05 were included in the meta-analysis. After the meta-analysis, associations in CpG-sites with FDR-adjusted *p* < 0.05 were considered statistically significant. Exploratory meta-analyses relating to the hypothesis for secondary birth exposures were conducted in the NFBC cohorts for dichotomous SGA-status, as well as birth weight SD and gestational age as continuous variables.

Demographic and clinical data were analyzed with SPSS (version 27) and methylation outcome data analyses and meta-analyses were performed with R statistical environment.

## Results

### Cohort profiles

Characteristics of our study subjects from the discovery and replication cohorts are described in Table 1. The mean age of our discovery cohort subjects was 22 years. In our validation cohorts, ages ranged from newborn (cord blood, PREDO), early adolescence (mean age of 12 years, GLAKU), adolescence (mean age of 16 years, NFBC1986), and adulthood (mean ages of 31 years and 46 years, NFBC1966).

**Table 1:** Descriptive statistics for the EWAS from the discovery and follow up cohorts.

	HeSVA VLBW	HeSVA controls	PREDO	GLAKU	NFBC-1986	NFBC-1966 (2004)	NFBC-1966 (2014)
Number of participants	157	161	817	236	510	722	705
Sex (men/women)	68/89	64/97	433/384	121/115	241/269	317/405	308/397
Age (mean (SD); years)	22.4 (2.1)	22.5 (2.2)	0^a^	12.3 (0.50)	16.1 (0.36)	31.0 (0.33)	46.3 (0.43)
Birth weight (mean (SD); grams)	1120 (220)	3580 (460)	3620 (606)	3570 (472)	3608 (475)	3515 (506)	3510 (508)
Gestational age (mean (SD); weeks)	29.1 (2.2)	40.2 (1.2)	39.7 (1.8)	40.0 (1.3)	39.6 (1.3)	40.2 (1.8)	40.1 (1.8)
Birth weight SD from population (mean (SD); units)	−1.3 (1.5)	0.01 (1.0)	0.02 (1.1)	−0.00(1.0)	0.2 (1.0)	−0.1 (1.1)	−0.1 (1.1)
Maternal smoking during pregnancy (*n*, %)	30 (19.1%)	26 (16.1%)	34 (4.2%)	20 (8.5%)	107 (21.0%)	149 (20.6%)	143 (20.3%)
VLBW (*n*, %)	157 (49.4%)	0 (0%)	4 (0.5%)	0 (0%)	0 (0%)	1 (0.1%)	1 (0.1%)
SGA (*n*, %)	51 (33%)	0 (0%)	13 (1.6%)	2(0.8%)	6 (1.2%)	24 (3.4%)	24 (3.4%)

*VLBW* very low birth weight, <1500 g.

*SGA* small for gestational age, <−2 SD.

^a^neonatal samples from cord blood.

### Epigenome wide association study (EWAS) of the discovery cohort

**Fig. 1:**
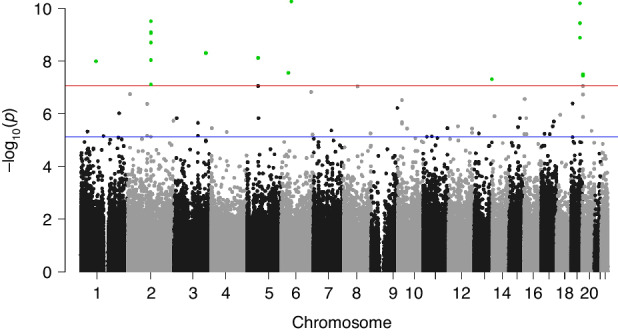
Manhattan plot of the epigenome-wide association study for the discovery cohort. The analysis was adjusted for sex, age, cell type proportions, maternal smoking during pregnancy, and parental socioeconomic status. The x-axis is the chromosomal position, and the y-axis is the level of statistical significance on a −log_10_-scale. The blue line represents the threshold for experiment wide significance at *p* = 7.6 × 10^−6^. CpGs highlighted in green represent sites with an epigenome wide significance of *p* < 9 × 10^−8^ (red line). The thresholds are based on previous recommendations.^38,39^

**Fig. 2:**
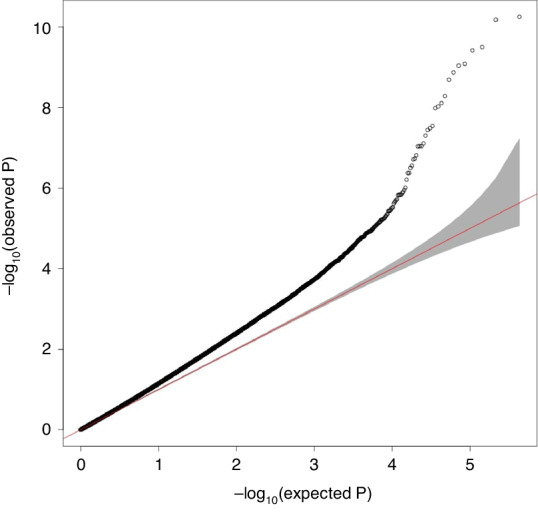
Quantile-quantile plot of the distribution. The observed association *p* values are plotted against the expected null distribution for the discovery analysis.

We identified 66 differentially methylated CpGs that were associated with birth at VLBW with a significance of FDR-adjusted *p* < 0.05 (Table 2). A Manhattan plot of the results is shown in Fig. 1 and a QQ-plot in Fig. 2. The QQ-plot has a lambda value of 1.16, but in EWAS such values are common and may not reflect true inflation.^37^ Seventeen CpGs were differentially methylated using the epigenome-wide *p* value thresholds for statistical significance of *p* < 9 × 10^-8^ and 23 with *p* < 2.44 × 10^-7^, as suggested previously.^38,39^ The CpG-site with the lowest *p* value in our study is cg21246516 (*p* < 5.8 × 10^-11^), located in an intergenic region between *TINAG* (dist = 413,877 bp) and *FAM83B* (dist = 42,693 bp) and is hypermethylated in those with VLBW. Our top DMPs localized to the genomic regions of GLI2, SKIDA1, HIF3A, EBF4, and SOCS1.

**Table 2:** Top 66 differentially methylated positions sorted in descending order by *p* values between VLBW subjects and normal birth weight controls showing probe names, chromosomes, and chromosomal positions, methylation differences between groups, *p* values, and gene locations at FDR < 0.05.

Probe	Chr	Start	End	Beta value	Standard error	*p* value	Gene or nearest gene	Location
cg21246516	6	54668827	54668876	−1.95E-02	2.87E-03	5.58E-11	TINAG(dist = 413,877), FAM83B (dist = 42,693)	intergenic
cg16672562	19	46801672	46801721	1.03E-01	1.51E-02	6.58E-11	HIF3A	ncRNA_UTR5
**cg00637745**	2	121497285	121497334	−9.22E-02	1.42E-02	3.15E-10	LOC84931 (dist = 273,360), GLI2 (dist = 57,533)	intergenic
cg27146050	19	46801508	46801557	3.39E-02	5.23E-03	3.78E-10	HIF3A	ncRNA_ intronic
**cg13872898**	2	121498145	121498194	−5.72E-02	9.01E-03	8.18E-10	LOC84931 (dist = 274,220), GLI2 (dist = 56,673)	intergenic
**cg20219891**	2	121496876	121496925	−8.24E-02	1.30E-02	9.06E-10	LOC84931 (dist = 272,951), GLI2 (dist = 57,942)	intergenic
cg22891070	19	46801642	46801691	8.10E-02	1.29E-02	1.34E-09	HIF3A	ncRNA_UTR5
**cg17870997**	2	121498522	121498571	−6.96E-02	1.12E-02	2.03E-09	LOC84931 (dist = 274,597), GLI2 (dist = 56,296)	intergenic
cg01721313	3	169751610	169751659	2.33E-02	3.86E-03	5.16E-09	SEC62 (dist = 35,449), GPR160 (dist = 4076)	intergenic
cg07593523	5	58082801	58082850	−4.21E-02	7.08E-03	7.74E-09	RAB3C	intronic
cg07133097	2	121497539	121497588	−2.13E-02	3.60E-03	9.37E-09	LOC84931 (dist = 273,614), GLI2 (dist = 57,279)	intergenic
cg11188223	1	79658904	79658953	4.64E-02	7.88E-03	1.03E-08	ELTD1 (dist = 186,409), NONE (dist = NONE)	intergenic
cg25325512	6	37142171	37142220	3.02E-02	5.29E-03	2.85E-08	PIM1	UTR3
cg24263062	20	2730191	2730240	−6.18E-02	1.09E-02	3.24E-08	EBF4	intronic
cg13518079	20	2675024	2675073	−6.93E-02	1.22E-02	3.59E-08	EBF4	intronic
cg11668844	13	113655623	113655672	1.60E-02	2.85E-03	4.98E-08	MCF2L	intronic
**cg14311362**	2	121498763	121498812	−6.73E-02	1.22E-02	7.79E-08	LOC84931 (dist = 274,838), GLI2 (dist = 56,055)	intergenic
cg16987982	5	58082727	58082776	−3.66E-02	6.67E-03	9.03E-08	RAB3C	intronic
cg14959908	20	2736835	2736884	−2.91E-02	5.30E-03	9.06E-08	EBF4	intronic
cg13213009	8	74903712	74903761	4.08E-02	7.45E-03	9.16E-08	LY96	exonic
cg00647262	6	157746535	157746584	−1.52E-02	2.82E-03	1.53E-07	TMEM242 (dist = 1282), ZDHHC14 (dist = 55,973)	intergenic
cg15374751	2	11925224	11925273	−1.80E-02	3.38E-03	1.86E-07	LPIN1	intronic
cg05857996	20	2675418	2675467	−5.49E-02	1.03E-02	1.93E-07	EBF4	intronic
cg01352090	16	4103534	4103583	1.34E-02	2.55E-03	2.77E-07	ADCY9	intronic
cg03610228	10	21799347	21799396	4.03E-02	7.70E-03	3.13E-07	C10orf114 (dist = 13,134), SKIDA1 (dist = 3013)	intergenic
cg25637722	19	6464034	6464083	1.24E-02	2.39E-03	4.21E-07	CRB3	upstream
cg19642470	2	102868023	102868072	2.55E-02	4.92E-03	4.22E-07	IL1RL2 (dist = 12,212), IL1RL1 (dist = 59,890)	intergenic
cg26581729	9	139939793	139939842	2.01E-02	3.94E-03	6.16E-07	NPDC1	intronic
cg16545821	1	204118841	204118890	6.62E-03	1.32E-03	9.71E-07	ETNK2	exonic
cg16071681	18	19925526	19925575	−2.40E-02	4.82E-03	1.11E-06	GATA6 (dist = 143,035), CTAGE1 (dist = 67,989)	intergenic
cg12816876	14	32363454	32363503	−1.76E-02	3.55E-03	1.24E-06	NUBPL (dist = 33,025), ARHGAP5-AS1 (dist = 181,122)	intergenic
cg05825244	20	2730488	2730537	−9.52E-02	1.93E-02	1.32E-06	EBF4	exonic
cg04661436	15	79169208	79169257	−1.80E-02	3.66E-03	1.46E-06	MORF4L1	intronic
cg09645336	5	60458808	60458857	1.91E-02	3.89E-03	1.46E-06	SMIM15	upstream
cg27306243	3	15902308	15902357	1.14E-02	2.33E-03	1.48E-06	ANKRD28 (dist = 1255), MIR563 (dist = 12,921)	intergenic
cg10784813	16	11348629	11348678	−1.80E-02	3.66E-03	1.49E-06	SOCS1	UTR3
cg09718582	2	240751197	240751246	−2.44E-02	5.02E-03	1.87E-06	LOC150935 (dist = 28,841), MIR4786 (dist = 131,186)	intergenic
cg21256649	17	66507611	66507660	1.32E-02	2.72E-03	1.97E-06	PRKAR1A	upstream
cg04707519	10	21799314	21799363	4.08E-02	8.43E-03	2.12E-06	C10orf114 (dist = 13,101), SKIDA1 (dist = 3046)	intergenic
cg14091208	3	128722580	128722629	1.86E-02	3.85E-03	2.24E-06	EFCC1	intronic
cg04714110	10	21799094	21799143	2.11E-02	4.39E-03	2.44E-06	C10orf114 (dist = 12,881), SKIDA1 (dist = 3266)	intergenic
cg20995304	12	48196168	48196217	2.08E-02	4.37E-03	3.00E-06	HDAC7	intronic
cg25098174	17	61512572	61512621	1.05E-02	2.20E-03	3.02E-06	CYB561	exonic
cg08362102	15	69087775	69087824	1.40E-02	2.94E-03	3.23E-06	ANP32A	intronic
cg03554158	4	4323322	4323371	6.10E-03	1.29E-03	3.53E-06	ZBTB49	UTR3
cg01900413	11	128419357	128419406	1.38E-02	2.92E-03	3.61E-06	ETS1	intronic
cg08415998	10	50602615	50602664	−3.25E-02	6.88E-03	3.70E-06	DRGX	intronic
cg25463779	12	124773237	124773286	−1.39E-02	2.94E-03	3.70E-06	ZNF664-FAM101A	intronic
cg22171829	7	95225471	95225520	2.79E-02	5.97E-03	4.36E-06	PDK4	exonic
cg06260709	20	48626573	48626622	−2.62E-02	5.61E-03	4.50E-06	SNAI1 (dist = 21,153), LINC00651 (dist = 30,622)	intergenic
cg14091978	1	34652561	34652610	−2.12E-02	4.54E-03	4.82E-06	C1orf94	intronic
cg16744741	4	82125976	82126025	2.24E-02	4.82E-03	4.95E-06	PRKG2	exonic
cg09854317	12	123754187	123754236	8.74E-03	1.88E-03	5.30E-06	CDK2AP1	intronic
cg14566475	8	144295010	144295059	1.62E-02	3.51E-03	5.64E-06	GPIHBP1	upstream
cg01154353	13	44953859	44953908	−8.58E-03	1.86E-03	5.69E-06	SERP2	intronic
cg11024682	17	17730045	17730094	1.23E-02	2.66E-03	5.79E-06	SREBF1	intronic
cg15020801	17	46022809	46022858	1.66E-02	3.60E-03	6.01E-06	PNPO	intronic
cg27063372	16	616809	616858	2.17E-02	4.71E-03	6.03E-06	NHLRC4	upstream
cg04635642	16	12183890	12183939	1.66E-02	3.61E-03	6.09E-06	SNX29	intronic
cg18132363	6	166260523	166260572	2.92E-02	6.34E-03	6.18E-06	PDE10A (dist = 184,935), LINC00473 (dist = 76,964)	intergenic
cg23417875	2	102313070	102313119	1.21E-02	2.65E-03	6.77E-06	RFX8 (dist = 221,905), MAP4K4 (dist = 1046)	intergenic
cg01886524	3	128225825	128225874	−2.44E-02	5.32E-03	6.92E-06	LOC90246	upstream
cg06814792	1	120174571	120174620	1.39E-02	3.05E-03	7.00E-06	ZNF697	intronic
cg13326175	11	45718185	45718234	−1.85E-02	4.06E-03	7.32E-06	CHST1 (dist = 30,979), LOC100507384 (dist = 25,624)	intergenic
cg14123232	2	121494702	121494751	1.33E-02	2.92E-03	7.57E-06	LOC84931 (dist = 270,777), GLI2 (dist = 60,116)	intergenic
cg05895618	11	19222346	19222395	1.32E-02	2.89E-03	7.60E-06	CSRP3	intronic

Adjusted for sex, age, maternal smoking, parental education level, and blood cell type proportions. The threshold of *p* < 9 × 10^-8^ is marked by a thick horizontal line. The CpGs validated in a meta-analysis combining the follow-up cohorts using continuous birth weight as the exposure are in bold. A negative beta indicates lower methylation in normal birth weight individuals.

### Meta-analysis in the validation cohorts

**Fig. 3:**
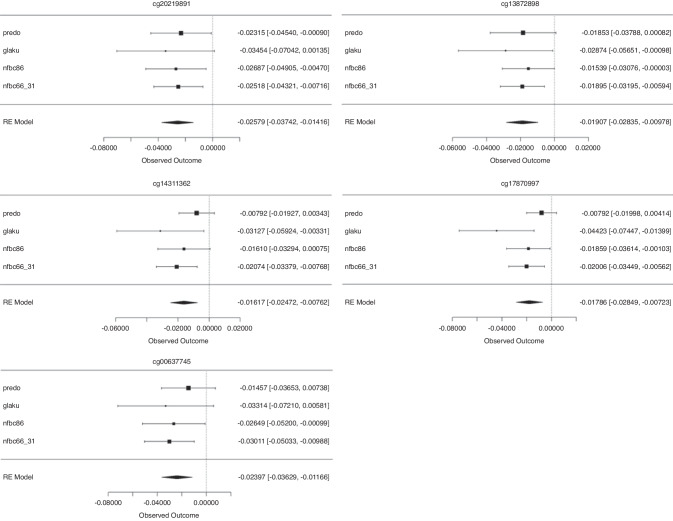
Forest plots showing meta-analysis of significant DNA methylation betas. Meta-analysis of DNA methylation betas is shown for birth weight (kg) at CpGs across four studies (PREDO, GLAKU, NFBC1986, and NFBC1966) with 95% confidence intervals after FDR-correction by Benjamini-Hochberg method at level 0.05. Adjusted for age, sex, maternal smoking during pregnancy, parental education, technical covariates, and cell type proportions.

We checked whether the top 66 differentially methylated CpG-sites with FDR-adjusted *p* < 0.05 could be validated in four independent cohorts with an overall age ranging between 0 and 31 years using continuous birth weight as the exposure. We used linear regression analyses and estimated regression coefficients and SEs, describing the linear relationship between birth weight and methylation adjusting for similar factors as in the discovery cohort’s analysis: blood cell type proportions, age, sex, maternal smoking during pregnancy, and parental socioeconomic status (see Supplementary Table 1–5). We then ran meta-analyses across the 66 top-associated CpG-sites to assess whether findings from the HeSVA discovery cohort could be followed up and validated in the cohorts of PREDO, GLAKU, NFBC1986, and NFBC1966. This resulted in five CpGs that were hypermethylated both in the VLBW group and with lower birth weight at FDR-adjusted *p* < 0.05 (cg20219891, cg13872898, cg00637745, cg14311362 and cg17870997). The forest plots for the meta-analysis of the validating sites are presented in Fig. 3.

### Secondary exposure analyses

Exploratory analyses over the 66 top hits from HeSVA were conducted in NFBC1986 and NFBC1966 with being born preterm (<37 gestational weeks) and SGA as dichotomous exposures, and gestational age and birth weight SD as continuous variables. 41 CpGs were differentially methylated with these secondary exposures, with overlapping sites for the primary birth weight exposure. The results of the secondary exposure analyses are described in Supplementary Table 5.

No single CpG of the discovery cohort’s 66 sites was differentially methylated in all cohorts even though they were validated in the meta-analysis. If we only observe samples from beyond infancy, however, seven CpGs show differential methylation with exposures varying among birth weight, birth weight SD, preterm-status and SGA-status. These sites were located in intergenic (*GLI2*, *CTAGE1*), intronic, and exonic (*EBF4*) as well as in UTR3’-regions (*SOCS1*). The DMPs and their respective cohorts and exposures are described in Supplementary Table 6.

We further conducted exploratory meta-analyses over NFBC1986 and NFBC1966 with being born preterm as the exposure. One CpG (cg14123232, intergenic region with nearest gene being *GLI2*), was differentially methylated in the meta-analysis at nominal significance (*p* = 0.044), but this did not survive FDR-correction. Forest plots for prematurity in NFBC1986 and NFBC1966 for the validating sites from the main meta-analysis with birth weight as the exposure, and for the nominally significant site cg14123232 are presented in Supplementary Fig. 1.

## Discussion

We hypothesized that there are blood DNAm differences between young adults born preterm at VLBW and term controls. In the HeSVA-cohort, we identified 66 differentially methylated CpG-sites between 157 VLBW individuals and 161 term controls in early adulthood at mean age of 22 years. Our top DMPs localize to the genomic regions: *GLI2*, *SKIDA1*, *HIF3A*, *EBF4,* and *SOCS1*. The genes have been previously associated with early life development^18,40^ and maturation^41^ as well as with metabolic pathways^42,43^ suggesting relevant genomic sites possibly connecting adverse health consequences and severe early life exposure.

We further hypothesized that differences seen associating with VLBW could be validated in unrelated cohorts regarding birth weight. Of the 66 sites, five (all annotating to an intergenic region close to *GLI2*) were validated in a meta-analysis of four independent cohorts using continuous birth weight as main exposure, with subjects aged 0–31 years. Our findings may represent persisting changes in the epigenome that could partly explain differences in health outcomes between individuals born at VLBW and at term. We further explored secondary birth exposures, such as SGA, gestational age, and birth weight SD, and their association with DNAm differences.

The association between preterm birth or low birth weight and later health outcomes has been suggested to be in part mediated by epigenetics. Differences in methylation have been found between VLBW individuals and term controls but their persistence has remained unclear.^44^ The epigenetic signature of preterm birth at VLBW is present in adult life in our study. This makes preterm birth at VLBW one of the few early life exposures where such a signature can be replicably observed after adolescence. Considering the large number of VLBW or preterm individuals globally, identifying molecular mechanisms between early life exposures and later outcomes is important. Epigenetic differences have been studied in individuals with different degrees of prematurity, usually graded by birth weight or gestational age. Genes annotating to DMPs that have been reported in connection with prematurity associate with the immune system,^45^ neurologic development,^46^ cellular differentiation and proliferation,^47^ metabolism (e.g., IGF2),^20,48^ fetal development, and birth weight.^49^ Significant DMPs are also seen in intergenic regions that may act as binding sites for transcription factors. The most significant and compelling CpG-sites of our study are located within or near genes associating with fetal development (*GLI2*),^50^ organogenesis (*SKIDA1*),^51^ insulin resistance and hypoxia response (*HIF3A*),^52^ neural development and B-cell maturation (*EBF4*),^53^ and immunological development (*SOCS1*).^40^

Our most robust finding is the validation of five DMPs in a meta-analysis of four independent cohorts, suggesting an association between birth weight and DNAm over a wide age range, which may represent long-lasting epigenetic imprint of prenatal factors contributing to birth weight. VLBW or lower birth weight individuals had higher methylation in CpGs cg00637745, cg13872898, cg20219891, cg17870997, and cg14311362. These were validated at FDR < 0.05 in our meta-analysis and are in an intergenic region closest to *GLI2*. A meta-analysis investigating associations between birth weight and cord blood DNAm (*n* = 8825) in term subjects found differentially methylated CpGs nearest to *GLI2* in childhood (*n* = 2756), adolescence (*n* = 2906), and adulthood (*n* = 1616).^19^ The five CpGs from our study are present in the meta-analysis which, too, reports a negative association between methylation levels and birth weight at these sites. Cameron et al. report hypermethylation in one CpG site associating with GLI2 (cg20219891), which is one of the sites seen in our discovery cohort and validation meta-analysis.^22^ Differential methylation of *GLI2* has been associated with gestational age in a meta-analysis in cord blood.^18^ None of the five validated CpGs from our study examining birth weight were reported as differentially methylated.^18^ *GLI2* is a gene in chromosome 2 encoding the protein *Gli family zinc finger 2*, which is involved in intracell signaling via the Sonic Hedgehog pathway.^50^ *GLI2* is also involved in neural tube development, and embryogenesis, as well as being a potential oncogene.^54^

Three CpGs (cg03610228, cg04707519, and cg04714110) associating with *SKIDA1* were among our discovery cohort’s top hits with a positive association between DNAm levels and VLBW status. These sites, however, were not validated in the meta-analysis examining continuous birth weight. A positive association between DNAm levels and gestational age has previously been reported for cg03610228, cg04707519, and cg04714110 in infants.^55^ A positive association between DNAm levels and birth weight for cg04714110 has been observed in cord blood.^19^ *SKIDA1* is a gene in chromosome 10 encoding the protein *SKI/DACH domain containing 1* (also known as *DLN-1; C10orf140*), which has a predicted localization in the nucleus and cytosol, which may reflect molecular trafficking between the two. It has been reported to have a role in autoimmune disorders.^56^ Animal models suggest involvement in organogenesis and brain maturation,^51^ and gastrulation and neurulation.^41^ The importance of *SKIDA1* is highlighted by *SKIDA1* knockout mice being subviable.^57^

We observed hypomethylation with VLBW of three epigenome-wide CpG-sites (cg27146050, cg16672562, and cg22891070) annotated to the gene *HIF3A*. These sites are also differentially methylated in NFBC1966 at 31 years of age, although they were not validated in the subsequent meta-analysis. Cameron et al. also report hypomethylation in three sites associating with *HIF3A* in 28-year-old VLBW individuals when compared to term controls.^22^ Our sites are among these. Differential methylation of *HIF3A* has been associated with gestational age in cord blood.^18,58^ *HIF3A* is a gene in chromosome 19 encoding the protein *hypoxia inducible factor 3 subunit alpha*, which is involved in regulating adaptive processes linked to low oxygen and metabolism.^59^ Such processes play roles in the regulation of glucose metabolism and cardiometabolic risk factors.^60^ *HIF3A* is largely expressed in placental, fat and spleen tissues.^61^ Hypermethylation of *HIF3A* has been linked to weight and BMI gain^42,62^ and to insulin resistance in gestational and type 2 diabetes^52^ and adipose tissue differentiation and dysfunction.^63^ Cord blood *HIF3A* methylation levels have also been associated with pre-eclampsia,^64^ and elevated blood pressure in childhood,^65^ suggesting a possibility of being a mediating element of cardiovascular risk factors. Our findings are in concordance with previously reported increased cardiometabolic risk factors in VLBW and ELBW adults.^2,66^

A fourth gene of interest is *EBF4*, as four intronic CpGs (cg24263062, cg13518079, cg14959908, and cg05857996) and one exonic CpG (cg05825244) are among our top hits with hypermethylation in the VLBW group and lower birth weight. The *EBF4*-associated CpGs are present in all our replication cohorts excluding infancy with varying early life exposures (Supplementary Table 7). *EBF4* is a gene in chromosome 20 encoding the protein *early B-cell factor transcription factor 4*, which has been associated with neural development and B-cell maturation.^53^ Few publications regarding methylation of *EBF4* and biological development or growth have been published, but *EBF4* methylation has been associated with BMI in adults.^43^ Differential methylation of *EBF4* has been positively associated with gestational age, also in adolescence,^18^ but in opposite direction to our study. Cameron et al. report four CpG-sites associating with *EBF4* being differentially methylated between VLBW participants and term controls in 28-year-olds^22^; four of these sites (cg24263062, cg05857996, cg13518079, and cg05825244) overlap with our results.

The CpG (cg10784813) associating with *SOCS1* is hypermethylated in VLBW, which is in line with Küpers et al. reporting a negative association between DNAm levels and birth weight in cg10784813.^19^ Alongside *GLI2* it is one of the few DMPs present in all our time points from adolescence onwards, albeit with varying exposures making its validity uncertain (Supplementary Table 7). *SOCS1* is a gene in chromosome 16 encoding the protein *suppressor of cytokine signaling 1*, which deals with inflammatory reactions and is particularly present in lymphatic tissue.^67^ *SOCS1* deficiency is associated with perinatal lethality, presumably due to its role in immunology.^40^ Differential methylation of *SOCS1* in cord blood has a positive association with gestational age reported by Merid et al.^18^ who also report replicating their finding in an independent sample by Hannon et al.^13^

Previous meta-analyses concerning birth weight and gestational age have found DMPs in the same regions as in our study,^18,19,68^ but most studies have been conducted in neonates and from cord blood. In the meta-analysis by Küpers et al.^19^ 8170 CpGs related to birth weight were identified, and of our top 66 CpG hits, there are 16 overlapping sites with the same directionality of methylation. In a meta-analysis assessing DNAm and gestational age in non-complicated births^18^ 8899 CpGs were identified but only 10 CpGs overlapped with our study with the same directionality. They also report results for all births (complicated and non-complicated) with 17,095 CpGs related to gestational age, but there is considerable heterogeneity in this group. The overlap with the ”all births” model and our study is 25 CpGs. The meta-analyses mentioned here are mainly based on cord blood and may not be entirely representative of our study with Finnish adult participants.

Individuals born at VLBW have been shown to have several DMPs compared to term controls, but the results vary considerably across studies. A study of 12 individuals born at gestational age <31 weeks and 12 matched term controls identified 1,555 DMPs in cord blood at birth, most of which resolved by the age of 18.^45^ A study of 40 VLBW infants showed DNAm differences in buccal samples in *IGF2* and *FKBP5* at birth but they resolved after first year of life, which may reflect tissue-specific mechanisms and may not be comparable to studies based on blood.^69^ Another similar-sized study showed hypomethylation of *PLAGL1* in VPT individuals at birth and at discharge.^70^ A small study comparing VLBW and term children at 12 months of age showed methylation differences in *SLC6A3*.^71^ *IGF2* has been identified as a gene that is differentially methylated from VLBW infants up to 20-year-olds.^20^ A recent study by Cameron et al. compared DNAm between 109 VLBW individuals and 51 term controls using heel prick spot blood samples from birth, and venous blood at 28 years of age from 215 VLBW individuals and 96 term controls.^22^ They report 16,400 CpG-sites differing between the groups at birth, but at the age of 28 years, only twelve were differentially methylated after FDR-correction. It is of particular note, that of these twelve CpG-sites, eight were differentially methylated in our study as well. A study comparing ELBW individuals to normal birth weight individuals in adulthood found buccal cell DNAm differences in 1,354 loci in 694 genes in men, but only two loci in women.^21^ Epigenome-wide differences have also been studied in individuals born with a less severe exposure of LBW. Fewer DMPs tend to be identified when comparing LBW individuals to term controls than when studying VLBW individuals, which suggests that there might be a threshold- or dose-effect phenomenon connecting the degree of prematurity and methylation. Simpkin et al.^72^ aimed to see whether long-term changes to the epigenome could be identified by sampling 1018 mostly term-born children at birth, and at ages 7 and 17, but they could not detect persistent changes in the epigenome associated with birth weight. A study by Madden et al. reports only one significant DNAm CpG-site (cg00966482) associated with birth weight (n of LBW = 84) in adults with a cohort mean age of 29.5.^73^ Aside from the CpGs associating with *GLI2*, *EBF4,* and *HIF3A* reported by Cameron et al., none of the previously reported CpGs or genes from studies with VLBW or LBW participants were significant in our study, which underlines the heterogeneity of the results and the need for large-scale meta-analyses. Methylation patterns tend to change during early development, and reported methylation differences between preterm and term individuals tend to largely resolve, which may reflect metabolic adjustments during maturation. Persisting epigenetic differences may reflect long-lasting differences in metabolism, but this requires more study.

We aimed to explore whether DNAm could be linked to SGA or prematurity, as well as examining gestational age and birth weight as continuous variables. Based on our findings, the epigenetic signatures of these phenomena overlap, which is to be expected, as the clinical phenotypes themselves overlap. However, being SGA could associate with a separate DNAm identifier, as some adverse adult health outcomes of preterm birth may be more apparent in individuals born SGA. Our meta-analysis of preterm participants in NFBC1986 and NFBC1966 had too few SGA participants (*n* = 0 for NFBC1986 and *n* = 25 for NFBC1966) to reach conclusions. No definite conclusions about the other secondary exposures could be drawn from these results either. Therefore, our results need to be replicated in a larger sample of preterm and preterm at VLBW subjects. Furthermore, when considering the effect of prematurity on DNAm, the nature of the exposure should be considered carefully as both gestational age and birth weight may have separate, yet often overlapping effects. Thus, we explored both gestational length and size at birth as exposures.

A major strength of our discovery cohort is the large number of VLBW participants in adulthood, as much of the past research is conducted in either infants or individuals with a less severe exposure. We also have access to four extensively phenotyped and characterized independent follow-up cohorts of varying ages, which we combined into a meta-analysis, to validate our findings. The age of our cohorts spans over decades of life with PREDO starting from birth and finishing with NFBC1966 in adulthood. Methylation patterns change rapidly during early life, which may explain why only few of the differentially methylated CpGs in the discovery cohort could be identified from newborn samples.

The age of our discovery cohort subjects at the time of measurement was relatively early in adulthood and it remains to be seen whether the epigenetic differences persist further into adulthood and what their relationship with future cardiovascular risk is. It should also be noted that contrary to the other birth cohorts, PREDO is built around examining risk factors for pre-eclampsia, which may introduce confounding. Even though our study spans a wide age range of participants, no longitudinal analysis was possible. Thus, DNAm differences seen in adulthood between VLBW and term individuals could be due to other biological differences that have accumulated during the participants’ life. Causality cannot be shown in this setting. Our results showed some inflation, which is often observed in EWAS.^37^

We aimed to investigate the DNAm differences between young adults born at VLBW and their term controls. We found 66 differentially methylated CpG-sites, which suggests a marker of preterm birth at VLBW present at young adulthood. We further saw several of these sites when examining birth weight in independent cohorts of varying ages validating our findings. While there is heterogeneity and noise between published studies, consistent and replicable gene regions where epigenetic signatures of early life events persist are starting to emerge. We believe this highlights the importance of collaboration and wider meta-analyses.

## Conclusions

In comparing young adults born preterm at low birth weight with controls born at term, we have discovered differentially methylated CpG-sites that are associated with birth weight in cohorts ranging from newborns to adulthood.

Our results support earlier findings and provide more robust evidence that epigenetic differences with regard to birth weight may remain even decades after preterm birth. Future studies should investigate whether our findings can be replicated in longitudinal cohorts and also continue the follow-up to see if the epigenetic differences show persistence in later life, and how these differences manifest phenotypically.

## Supplementary information

Supplementary material

## Data Availability

The datasets generated during and/or analyzed during the current study are available from the corresponding author on reasonable request.
