# Supplementary material for: Epigenetic signature of very low birth weight in young adult life
Source: Pediatr Res. 2024 Jun 19;97(1):229–38. doi: 10.1038/s41390-024-03354-6 (PMC11798856; doi:10.1038/s41390-024-03354-6)
Supplement: Supplementary file 1 — Supplementary material [file 41390_2024_3354_MOESM1_ESM.pdf]

Supplemental figure 1. Forest plots showing meta-analysis of DNA methylation betas for prematurity at chosen CpGs across NFBC1986 and with 95% Confidence Intervals. Adjusted for age, sex, maternal smoking during pregnancy, parental education, technical covariates and cell type proportions.

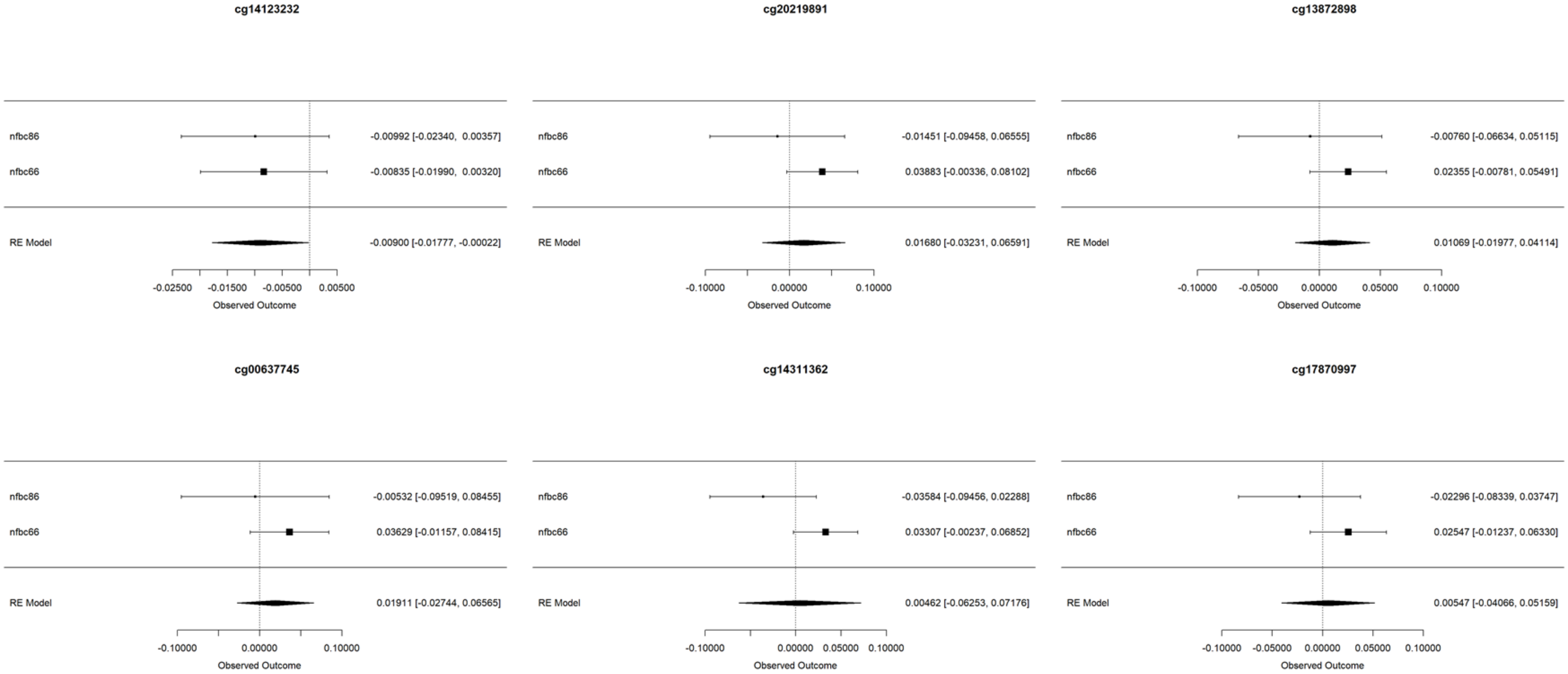

Supplemental table 1. Differentially methylated positions between VLBW and normal birth weight subjects in the discovery cohort (FDR-corrected p-value < 0.05) that were also differentially methylated (p-value < 0.05) in the PREDO cohort showing probe names, methylation differences between groups, adjusted p-values and FDRs, and gene locations. The CpGs observed in a meta-analysis combining the follow-up cohorts using continuous birth weight as the exposure are in bold. A negative beta indicates decreasing methylation with increasing birth weight.

Probe	Chr	Start	End	Discovery				Replication in PREDO				
				Beta value	SE	p-value	FDR	Beta value	SE	p-value	Gene or nearest gene	Location
**cg20219891**	2	121496876	121496925	-8.24E-02	1.30E-02	9.06E-10	6.48E-05	-2.31E-05	1.14E-05	4.18E-02	*GLI2* (dist=57942)	intergenic
cg03610228	10	21799347	21799396	4.03E-02	7.70E-03	3.13E-07	5.37E-03	1.50E-05	5.76E-06	9.50E-03	*SKIDA1* (dist=3013)	intergenic
cg04714110	10	21799094	21799143	2.11E-02	4.39E-03	2.44E-06	2.55E-02	1.65E-05	3.72E-06	9.92E-06	*SKIDA1* (dist=3266)	intergenic
cg01900413	11	128419357	128419406	1.38E-02	2.92E-03	3.61E-06	3.31E-02	8.67E-06	3.49E-06	1.32E-02	*ETS1*	intronic
cg22171829	7	95225471	95225520	2.79E-02	5.97E-03	4.36E-06	3.82E-02	8.11E-06	3.40E-06	1.74E-02	*PDK4*	exonic
cg15020801	17	46022809	46022858	1.66E-02	3.60E-03	6.01E-06	4.42E-02	9.19E-06	4.63E-06	4.77E-02	*PNPO*	intronic
cg01886524	3	128225825	128225874	-2.44E-02	5.32E-03	6.92E-06	4.76E-02	-6.23E-06	3.01E-06	3.85E-02	*LOC90246*	upstream

Supplemental table 2. Differentially methylated positions between VLBW and normal birth weight subjects in the discovery cohort (FDR-corrected p-value < 0.05) that were also differentially methylated (p < 0.05) in the GLAKU cohort showing probe names, chromosomes and chromosomal positions, methylation differences between groups, adjusted p-values and FDRs, and gene locations. The CpGs observed in a meta-analysis combining the follow-up cohorts using continuous birth weight as the exposure are in bold. A negative beta indicates decreasing methylation with increasing birth weight.

Probe	Chr	Start	End	Discovery in HeSVA				Replication in GLAKU				
				Beta value	SE	p-value	FDR	Beta value	SE	p-value	Gene or nearest gene	Location
**cg13872898**	2	121498145	121498194	-5.72E-02	9.01E-03	8.18E-10	6.48E-05	-2.87E-05	1.42E-05	4.38E-02	*GLI2* (dist=56673)	intergenic
**cg17870997**	2	121498522	121498571	-6.96E-02	1.12E-02	2.03E-09	1.09E-04	-4.42E-05	1.54E-05	4.59E-03	*GLI2* (dist=56296)	intergenic
cg24263062	20	2730191	2730240	-6.18E-02	1.09E-02	3.24E-08	9.93E-04	-3.40E-05	1.55E-05	2.89E-02	*EBF4*	intronic
**cg14311362**	2	121498763	121498812	-6.73E-02	1.22E-02	7.79E-08	1.96E-03	-3.13E-05	1.43E-05	2.95E-02	*GLI2* (dist=56055)	intergenic
cg14959908	20	2736835	2736884	-2.91E-02	5.30E-03	9.06E-08	1.96E-03	-1.45E-05	7.07E-06	4.12E-02	*EBF4*	intronic
cg16071681	18	19925526	19925575	-2.40E-02	4.82E-03	1.11E-06	1.59E-02	-1.78E-05	6.68E-06	8.37E-03	*CTAGE1* (dist=67989)	intergenic
cg05825244	20	2730488	2730537	-9.52E-02	1.93E-02	1.32E-06	1.77E-02	-4.95E-05	2.02E-05	1.52E-02	*EBF4*	exonic
cg10784813	16	11348629	11348678	-1.80E-02	3.66E-03	1.49E-06	1.77E-02	-1.28E-05	6.10E-06	3.75E-02	*SOCS1*	UTR3
cg14123232	2	121494702	121494751	1.33E-02	2.92E-03	7.57E-06	4.93E-02	1.34E-05	5.55E-06	1.63E-02	*GLI2* (dist=60116)	intergenic

Supplemental table 3. Differentially methylated positions showing CpGs, beta-values, standard errors, p-values and genomic locations of NFBC1986 sites that were significant and FDR < 0.05 in the discovery cohort as well as significant (p < 0.05) in the replication cohort. Analysed by linear regression models for birth weight adjusting for age, sex, maternal smoking during pregnancy, parental education, technical covariates and cell type proportions. The CpGs observed in a meta-analysis combining the follow-up cohorts using continuous birth weight as the exposure are in bold. A negative beta indicates decreasing methylation with increasing birth weight.

Probe	Discovery				NFBC 1986 (age: 16)			Gene or nearest gene	Location
	Beta	SE	p	FDR	Beta	SE	p		
**cg00637745**	-9.22E-02	1.42E-02	3.15E-10	4.06E-05	-2.65E-05	1.30E-05	4.23E-02	*GLI2* (dist=57533)	intergenic
**cg20219891**	-8.24E-02	1.30E-02	9.06E-10	6.48E-05	-2.69E-05	1.13E-05	1.79E-02	*GLI2* (dist=57942)	intergenic
**cg17870997**	-6.96E-02	1.12E-02	2.03E-09	1.09E-04	-1.86E-05	8.96E-06	3.85E-02	*GLI2* (dist=56296)	intergenic
cg07133097	-2.13E-02	3.60E-03	9.37E-09	3.65E-04	-1.07E-05	3.62E-06	3.14E-03	*GLI2* (dist=57279)	intergenic
cg24263062	-6.18E-02	1.09E-02	3.24E-08	9.93E-04	-1.89E-05	9.45E-06	4.67E-02	*EBF4*	intronic
cg16987982	-3.66E-02	6.67E-03	9.03E-08	1.96E-03	-1.70E-05	6.45E-06	8.73E-03	*RAB3C*	intronic
cg12816876	-1.76E-02	3.55E-03	1.24E-06	1.72E-02	-1.06E-05	3.18E-06	9.53E-04	*NUBPL* (dist=33025)	intergenic
cg04707519	4.08E-02	8.43E-03	2.12E-06	2.33E-02	2.41E-05	9.77E-06	1.39E-02	*SKIDA1*(dist=3046)	intergenic
cg04714110	2.11E-02	4.39E-03	2.44E-06	2.55E-02	7.81E-06	3.50E-06	2.60E-02	*SKIDA1* (dist=3266)	intergenic
cg18132363	2.92E-02	6.34E-03	6.18E-06	4.42E-02	1.08E-05	5.25E-06	3.94E-02	*LINC00473* (dist=76964)	intergenic

Supplemental table 4. Differentially methylated positions showing CpGs, beta-values, standard errors, p-values and genomic locations of NFBC1966 (31-year-olds) sites that were significant and FDR < 0.05 in the discovery cohort as well as significant (p < 0.05) in the replication cohort. Analysed by linear regression models for birth weight adjusting for age, sex, maternal smoking during pregnancy, parental education, technical covariates and cell type proportions. The CpGs observed in a meta-analysis combining the follow-up cohorts using continuous birth weight as the exposure are in bold. A negative beta indicates decreasing methylation with increasing birth weight.

Probe	Discovery				NFBC 1966 (age: 31)			Gene or nearest gene	Location
	Beta	SE	p	FDR	Beta	SE	p		
cg21246516	-1.95E-02	2.87E-03	5.58E-11	1.41E-05	-4.59E-06	2.09E-06	2.83E-02	*FAM83B* (dist=42693)	intergenic
**cg00637745**	-9.22E-02	1.42E-02	3.15E-10	4.06E-05	-3.01E-05	1.03E-05	3.64E-03	*GLI2* (dist=57533)	intergenic
**cg13872898**	-5.72E-02	9.01E-03	8.18E-10	6.48E-05	-1.89E-05	6.64E-06	4.43E-03	*GLI2* (dist=56673)	intergenic
**cg20219891**	-8.24E-02	1.30E-02	9.06E-10	6.48E-05	-2.52E-05	9.19E-06	6.32E-03	*GLI2* (dist=57942)	intergenic
cg22891070	8.10E-02	1.29E-02	1.34E-09	8.22E-05	1.57E-05	6.85E-06	2.22E-02	*HIF3A*	ncRNA_UTR5
**cg17870997**	-6.96E-02	1.12E-02	2.03E-09	1.09E-04	-2.01E-05	7.36E-06	6.62E-03	*GLI2* (dist=56296)	intergenic
cg07133097	-2.13E-02	3.60E-03	9.37E-09	3.65E-04	-7.29E-06	3.43E-06	3.38E-02	*GLI2* (dist=57279)	intergenic
**cg14311362**	-6.73E-02	1.22E-02	7.79E-08	1.96E-03	-2.07E-05	6.66E-06	1.93E-03	*GLI2* (dist=56055)	intergenic
cg18132363	2.92E-02	6.34E-03	6.18E-06	4.42E-02	8.79E-06	3.92E-06	2.51E-02	*LINC00473* (dist=76964)	intergenic

Supplemental table 5. Differentially methylated positions showing CpGs, beta-values, standard errors, p-values and genomic locations of NFBC cohort sites that were significant and FDR < 0.05 in the discovery cohort as well as significant (p < 0.05) in the replication cohort. Analysed by linear regression models adjusting for age, sex, maternal smoking during pregnancy, parental education, technical covariates and cell type proportions. The exposures are BWTSD = birth weight standard deviation as a continuous variable, GA = gestational age in weeks as a continuous variable, PT (prematurity, < 37 gestational weeks) dichotomously, SGA = small for gestational age (< -2 SD) dichotomously. The CpGs observed in a meta-analysis combining the follow-up cohorts using continuous birth weight as the exposure are in bold. A negative beta indicates decreasing methylation with increasing birth weight.

Probe	Discovery				NFBC 1966 (age: 46)				Gene or nearest gene	Location
	Beta	SE	p	FDR	Beta	SE	p	exposure		
cg16672562	1.03E-01	1.51E-02	6.58E-11	1.41E-05	1.22E-02	4.26E-03	4.16E-03	BWTSD	*HIF3A*	ncRNA_UTR5
cg27146050	3.39E-02	5.23E-03	3.78E-10	4.06E-05	5.13E-03	1.64E-03	1.80E-03	BWTSD	*HIF3A*	ncRNA_intronic
cg22891070	8.10E-02	1.29E-02	1.34E-09	8.22E-05	1.40E-02	4.65E-03	2.69E-03	BWTSD	*HIF3A*	ncRNA_UTR5
cg13518079	-6.93E-02	1.22E-02	3.59E-08	1.03E-03	-1.06E-02	4.20E-03	1.15E-02	BWTSD	*EBF4*	intronic
cg05857996	-5.49E-02	1.03E-02	1.93E-07	3.59E-03	-1.08E-02	5.26E-03	4.03E-02	BWTSD	*EBF4*	intronic
cg25637722	1.24E-02	2.39E-03	4.21E-07	6.70E-03	2.82E-03	1.07E-03	8.73E-03	BWTSD	*CRB3*	upstream
cg04707519	4.08E-02	8.43E-03	2.12E-06	2.33E-02	4.93E-03	2.43E-03	4.32E-02	BWTSD	*SKIDA1 (dist=3046)*	intergenic
cg22171829	2.79E-02	5.97E-03	4.36E-06	3.82E-02	3.77E-03	1.85E-03	4.21E-02	BWTSD	*PDK4*	exonic
cg16744741	2.24E-02	4.82E-03	4.95E-06	4.08E-02	3.81E-03	1.74E-03	2.85E-02	BWTSD	*PRKG2*	exonic
cg14566475	1.62E-02	3.51E-03	5.64E-06	4.42E-02	2.83E-03	1.08E-03	8.72E-03	BWTSD	*GPIHBP1*	upstream
cg04635642	1.66E-02	3.61E-03	6.09E-06	4.42E-02	4.26E-03	1.63E-03	8.97E-03	BWTSD	*SNX29*	intronic
**cg00637745**	-9.22E-02	1.42E-02	3.15E-10	4.06E-05	-5.24E-03	2.43E-03	3.15E-02	GA	*GLI2* (dist=57533)	intergenic
cg07133097	-2.13E-02	3.60E-03	9.37E-09	3.65E-04	-1.47E-03	7.22E-04	4.26E-02	GA	*GLI2* (dist=57279)	intergenic
**cg00637745**	-9.22E-02	1.42E-02	3.15E-10	4.06E-05	4.62E-02	2.22E-02	3.84E-02	PT	*GLI2* (dist=57533)	intergenic
**cg13872898**	-5.72E-02	9.01E-03	8.18E-10	6.48E-05	3.68E-02	1.69E-02	3.00E-02	PT	*GLI2* (dist=56673)	intergenic
**cg17870997**	-6.96E-02	1.12E-02	2.03E-09	1.09E-04	4.33E-02	1.97E-02	2.87E-02	PT	*GLI2* (dist=56296)	intergenic
cg07133097	-2.13E-02	3.60E-03	9.37E-09	3.65E-04	1.36E-02	6.51E-03	3.71E-02	PT	GLI2 (dist=57279)	intergenic

**cg14311362**	-6.73E-02	1.22E-02	7.79E-08	1.96E-03	3.96E-02	1.64E-02	1.58E-02	PT	*GLI2* (dist=56055)	intergenic
cg26581729	2.01E-02	3.94E-03	6.16E-07	9.43E-03	-1.91E-02	5.40E-03	4.35E-04	PT	*NPDC1*	intronic
cg10784813	-1.80E-02	3.66E-03	1.49E-06	1.77E-02	2.00E-02	7.68E-03	9.35E-03	PT	*SOCS1*	UTR3
cg03554158	6.10E-03	1.29E-03	3.53E-06	3.31E-02	-5.28E-03	1.81E-03	3.65E-03	PT	*ZBTB49*	UTR3
cg06260709	-2.62E-02	5.61E-03	4.5E-06	3.86E-02	1.88E-02	8.03E-03	1.97E-02	PT	*SNAI1* (dist=21153)	intergenic
cg14123232	1.33E-02	2.92E-03	7.57E-06	4.93E-02	-1.28E-02	5.68E-03	2.45E-02	PT	*GLI2* (dist=60116)	intergenic
cg16672562	1.03E-01	1.51E-02	6.58E-11	1.41E-05	-4.72E-02	2.26E-02	3.72E-02	SGA	*HIF3A*	ncRNA_UTR5
cg27146050	3.39E-02	5.23E-03	3.78E-10	4.06E-05	-2.06E-02	8.61E-03	1.69E-02	SGA	*HIF3A*	ncRNA_intronic
cg22891070	8.10E-02	1.29E-02	1.34E-09	8.22E-05	-5.10E-02	2.14E-02	1.74E-02	SGA	*HIF3A*	ncRNA_UTR5
cg07593523	-4.21E-02	7.08E-03	7.74E-09	3.32E-04	2.41E-02	1.11E-02	3.04E-02	SGA	*RAB3C*	intronic
cg25325512	3.02E-02	5.29E-03	2.85E-08	9.41E-04	-2.64E-02	1.30E-02	4.27E-02	SGA	*PIM1*	UTR3
cg13518079	-6.93E-02	1.22E-02	3.59E-08	1.03E-03	6.03E-02	2.61E-02	2.14E-02	SGA	*EBF4*	intronic
cg04714110	2.11E-02	4.39E-03	2.44E-06	2.55E-02	-1.87E-02	6.66E-03	5.03E-03	SGA	*SKIDA1* (dist=3266)	intergenic
Probe	Discovery				NFBC 1966 (age: 31)				Gene or nearest gene	Location
	Beta	SE	p	FDR	Beta	SE	p	exposure		
cg27146050	3.39E-02	5.23E-03	3.78E-10	4.06E-05	3.72E-03	1.51E-03	1.40E-02	BWTSD	*HIF3A*	ncRNA_intronic
cg22891070	8.10E-02	1.29E-02	1.34E-09	8.22E-05	8.88E-03	3.14E-03	4.76E-03	BWTSD	*HIF3A*	ncRNA_UTR5
cg16545821	6.62E-03	1.32E-03	9.71E-07	1.44E-02	8.07E-04	3.67E-04	2.81E-02	BWTSD	*ETNK2*	exonic
cg25463779	-1.39E-02	2.94E-03	3.7E-06	3.31E-02	-2.00E-03	9.39E-04	3.39E-02	BWTSD	*ZNF664-FAM101A*	intronic
cg08362102	1.40E-02	2.94E-03	3.23E-06	3.14E-02	1.27E-03	5.66E-04	2.53E-02	GA	*ANP32A*	intronic
cg16071681	-2.40E-02	4.82E-03	1.11E-06	1.59E-02	1.80E-02	7.08E-03	1.13E-02	PT	*CTAGE1* (dist=67989)	intergenic
cg10784813	-1.80E-02	3.66E-03	1.49E-06	1.77E-02	2.44E-02	7.57E-03	1.34E-03	PT	*SOCS1*	UTR3
cg09718582	-2.44E-02	5.02E-03	1.87E-06	2.17E-02	2.31E-02	8.01E-03	4.08E-03	PT	*LOC150935,* (dist=28841)	intergenic
cg27146050	3.39E-02	5.23E-03	3.78E-10	4.06E-05	-1.74E-02	7.76E-03	2.49E-02	SGA	*HIF3A*	ncRNA_intronic
cg24263062	-6.18E-02	1.09E-02	3.24E-08	9.93E-04	4.16E-02	2.11E-02	4.90E-02	SGA	*EBF4*	intronic
cg13518079	-6.93E-02	1.22E-02	3.59E-08	1.03E-03	6.06E-02	2.94E-02	3.95E-02	SGA	*EBF4*	intronic

cg14959908	-2.91E-02	5.30E-03	9.06E-08	1.96E-03	2.31E-02	1.17E-02	4.87E-02	SGA	*EBF4*	intronic
cg05825244	-9.52E-02	1.93E-02	1.32E-06	1.77E-02	8.36E-02	3.66E-02	2.26E-02	SGA	*EBF4*	exonic
Probe	Discovery				NFBC 1986 (age: 16)				Gene or nearest gene	Location
	Beta	SE	p	FDR	Beta	SE	p	exposure		
**cg00637745**	-9.22E-02	1.42E-02	3.15E-10	4.06E-05	-1.31E-02	6.32E-03	3.89E-02	BWTSD	*GLI2* (dist=57533)	intergenic
**cg20219891**	-8.24E-02	1.30E-02	9.06E-10	6.48E-05	-1.33E-02	5.48E-03	1.59E-02	BWTSD	*GLI2* (dist=57942)	intergenic
**cg17870997**	-6.96E-02	1.12E-02	2.03E-09	1.09E-04	-9.98E-03	4.35E-03	2.21E-02	BWTSD	*GLI2* (dist=56296)	intergenic
cg07133097	-2.13E-02	3.60E-03	9.37E-09	3.65E-04	-4.91E-03	1.74E-03	4.95E-03	BWTSD	*GLI2* (dist=57279)	intergenic
cg25325512	3.02E-02	5.29E-03	2.85E-08	9.41E-04	4.55E-03	2.29E-03	4.75E-02	BWTSD	*PIM1*	UTR3
cg13518079	-6.93E-02	1.22E-02	3.59E-08	1.03E-03	-1.17E-02	5.68E-03	3.98E-02	BWTSD	*EBF4*	intronic
**cg14311362**	-6.73E-02	1.22E-02	7.79E-08	1.96E-03	-9.38E-03	4.14E-03	2.38E-02	BWTSD	*GLI2* (dist=56055)	intergenic
cg16987982	-3.66E-02	6.67E-03	9.03E-08	1.96E-03	-9.63E-03	3.27E-03	3.39E-03	BWTSD	*RAB3C*	intronic
cg16071681	-2.40E-02	4.82E-03	1.11E-06	1.59E-02	-4.35E-03	1.80E-03	1.59E-02	BWTSD	*CTAGE1* (dist=67989)	intergenic
cg12816876	-1.76E-02	3.55E-03	1.24E-06	1.72E-02	-5.87E-03	1.58E-03	2.27E-04	BWTSD	*NUBPL* (dist=33025)	intergenic
cg04707519	4.08E-02	8.43E-03	2.12E-06	2.33E-02	1.04E-02	4.82E-03	3.14E-02	BWTSD	*SKIDA1* (dist=3046)	intergenic
cg18132363	2.92E-02	6.34E-03	6.18E-06	4.42E-02	5.89E-03	2.61E-03	2.46E-02	BWTSD	*LINC00473* (dist=76964)	intergenic
cg05857996	-5.49E-02	1.03E-02	1.93E-07	3.59E-03	3.60E-02	1.78E-02	4.32E-02	PT	*EBF4*	intronic
cg26581729	2.01E-02	3.94E-03	6.16E-07	9.43E-03	-2.30E-02	9.94E-03	2.09E-02	PT	*NPDC1*	intronic
cg09645336	1.91E-02	3.89E-03	1.46E-06	1.77E-02	-2.69E-02	9.06E-03	3.14E-03	PT	*SMIM15*	upstream
cg04707519	4.08E-02	8.43E-03	2.12E-06	2.33E-02	-3.53E-02	1.69E-02	3.72E-02	PT	*SKIDA1* (dist=3046)	intergenic
cg04714110	2.11E-02	4.39E-03	2.44E-06	2.55E-02	-2.69E-02	8.28E-03	1.23E-03	PT	*SKIDA1* (dist=3266)	intergenic
cg20995304	2.08E-02	4.37E-03	3E-06	3.01E-02	-2.82E-02	1.33E-02	3.42E-02	PT	*HDAC7*	intronic
cg08362102	1.40E-02	2.94E-03	3.23E-06	3.14E-02	-1.81E-02	6.53E-03	5.89E-03	PT	*ANP32A*	intronic

cg16744741	2.24E-02	4.82E-03	4.95E-06	4.08E-02	-2.87E-02	1.03E-02	5.75E-03	PT	*PRKG2*	exonic
cg14566475	1.62E-02	3.51E-03	5.64E-06	4.42E-02	-3.22E-02	1.38E-02	1.98E-02	PT	*GPIHBP1*	upstream
cg11024682	1.23E-02	2.66E-03	5.79E-06	4.42E-02	-1.67E-02	8.05E-03	3.86E-02	PT	*SREBF1*	intronic
**cg00637745**	-9.22E-02	1.42E-02	3.15E-10	4.06E-05	1.04E-01	2.53E-02	4.93E-05	SGA	*GLI2* (dist=57533)	intergenic
**cg13872898**	-5.72E-02	9.01E-03	8.18E-10	6.48E-05	6.42E-02	1.63E-02	9.39E-05	SGA	*GLI2* (dist=56673)	intergenic
**cg20219891**	-8.24E-02	1.30E-02	9.06E-10	6.48E-05	9.33E-02	1.61E-02	1.25E-08	SGA	*GLI2* (dist=57942)	intergenic
**cg17870997**	-6.96E-02	1.12E-02	2.03E-09	1.09E-04	6.33E-02	2.48E-02	1.10E-02	SGA	*GLI2* (dist=56296)	intergenic
cg07133097	-2.13E-02	3.60E-03	9.37E-09	3.65E-04	1.98E-02	5.45E-03	3.16E-04	SGA	*GLI2* (dist=57279)	intergenic
cg24263062	-6.18E-02	1.09E-02	3.24E-08	9.93E-04	5.18E-02	2.43E-02	3.36E-02	SGA	*EBF4*	intronic
**cg14311362**	-6.73E-02	1.22E-02	7.79E-08	1.96E-03	6.17E-02	2.63E-02	1.96E-02	SGA	*GLI2* (dist=56055)	intergenic
cg15374751	-1.80E-02	3.38E-03	1.86E-07	3.59E-03	3.52E-02	1.04E-02	7.73E-04	SGA	*LPIN1*	intronic
cg05857996	-5.49E-02	1.03E-02	1.93E-07	3.59E-03	4.40E-02	2.06E-02	3.32E-02	SGA	*EBF4*	intronic
cg05825244	-9.52E-02	1.93E-02	1.32E-06	1.77E-02	9.94E-02	4.65E-02	3.32E-02	SGA	*EBF4*	exonic
cg10784813	-1.80E-02	3.66E-03	1.49E-06	1.77E-02	2.16E-02	8.53E-03	1.18E-02	SGA	*SOCS1*	UTR3
cg06260709	-2.62E-02	5.61E-03	4.5E-06	3.86E-02	4.18E-02	1.30E-02	1.38E-03	SGA	*LINC00651* (dist=30622)	intergenic
cg01886524	-2.44E-02	5.32E-03	6.92E-06	4.76E-02	1.61E-02	4.74E-03	7.24E-04	SGA	*LOC90246*	upstream

Supplemental table 6. CpGs, beta-values, standard errors, p-values and genomic locations of discovery and replication cohort sites that were significant ($p < 0.05$) and FDR < 0.05 across all cohorts beyond infancy (i.e. PREDO cohort with cord blood not included). Analysed by linear regression models for birth weight adjusting for age, sex, maternal smoking during pregnancy, parental education, technical covariates and cell type proportions. The exposures are BWT = birth weight as a continuous variable, BWTSD = birth weight standard deviation as a continuous variable, PTSGA = small for gestational age (< -2 SD) dichotomously. GLAKU cohort had continuous birth weight as the exposure. The CpGs observed in a meta-analysis combining the follow-up cohorts using continuous birth weight as the exposure are in bold. A negative beta indicates decreasing methylation with increasing birth weight.

Probe	Discovery cohort						GLAKU (12 years)			NFBC1986 (16 years)				NFBC1966 (31 years)			
	Beta	SE	p	FDR	Gene or nearest gene	Location	Beta	SE	p	Beta	SE	p	exposure	Beta	SE	p	exposure
**cg13872898**	-0.0572	0.009	8.18E-10	6E-05	*GLI2* (dist=56673)	intergenic	-2.9E-05	1.4E-05	0.044	0.0642	0.0163	9.4E-05	SGA	-1.89E-05	6.64E-06	0.004	BWT
**cg17870997**	-0.0696	0.011	2.03E-09	0.0001	*GLI2* (dist=56296)	intergenic	-4.4E-05	1.5E-05	0.005	-2E-05	9E-06	0.0385	BWT	-2.01E-05	7.36E-06	0.007	BWT
										-0.01	0.0044	0.0221	BWTSD				
										0.0633	0.0248	0.011	SGA				
cg24263062	-0.0618	0.011	3.24E-08	0.001	*EBF4*	intronic	-3.4E-05	1.6E-05	0.029	-2E-05	9E-06	0.0467	BWT	0.0416	0.0211	0.049	SGA
										0.0518	0.0243	0.0336	SGA				
**cg14311362**	-0.0673	0.012	7.79E-08	0.002	*GLI2* (dist=56055)	intergenic	-3.1E-05	1.4E-05	0.03	-0.009	0.0041	0.0238	BWTSD	-2.07E-05	6.66E-06	0.002	BWT
										0.0617	0.0263	0.0196	SGA				
cg16071681	-0.024	0.005	1.11E-06	0.0159	*CTAGE1* (dist=67989)	intergenic	-1.8E-05	6.7E-06	0.008	-0.004	0.0018	0.0159	BWTSD	0.018	0.00708	0.011	PT
cg05825244	-0.0952	0.019	1.32E-06	0.0177	*EBF4*	exonic	-5E-05	2E-05	0.015	0.0994	0.0465	0.0332	SGA	0.0836	0.0366	0.023	SGA
cg10784813	-0.018	0.004	1.49E-06	0.0177	*SOCS1*	UTR3	-1.3E-05	6.1E-06	0.038	0.0216	0.0085	0.0118	SGA	0.0244	0.00757	0.001	PT

Supplementary methods

In PREDO, the child's birth weight was extracted from the Medical Birth Register and in GLAKU from health care records. In PREDO, the covariates included child's gestational age at birth, sex and maternal smoking during pregnancy which were extracted from the Medical Birth Register, HILMO, medical records and education which was mother-reported. These data were derived from the Finnish Medical Birth Register (MBR), HILMO or medical records. In addition, we adjusted for estimated cord blood cell counts for seven cell types (nucleated red blood cells, granulocytes, monocytes, natural killer cells, B cells, CD4(+)T cells, and CD8(+)T cells) using the method of Bakulski et al. which is incorporated in the R-package *minfi*. For PREDO, of the 1079 singleton live births, 963 cord blood samples were available for EWAS (epigenome-wide association study).

In GLAKU, the covariates included the child's sex, which came from medical records, and maternal smoking during pregnancy and level of education, which were reported by the mother, and the child's age was recorded at blood sampling. For GLAKU, peripheral venous blood, stored at -80°C, was obtained from 239 subjects for EWAS. DNA was bisulphite-converted using the EZ-96 DNA Methylation kit (Zymo Research, Irvine, CA). Genome-wide methylation profiling was conducted using the Illumina Infinium MethylationEPIC arrays (Illumina Inc., San Diego, CA) according to the manufacturer's protocol. Methylation analyses were performed at the Max Planck Institute of Psychiatry in Munich, Germany. After quality control, 236 individuals were included in the EWAS. The arrays were scanned using the iScan System (Illumina Inc., San Diego, CA).

In the PREDO and GLAKU follow up cohorts DNA was bisulphite-converted using the EZ-96 DNA Methylation kit (Zymo Research, Irvine, CA). After a quality control pipeline using the R-package *minfi*, the number of subjects available for analysis was 817. Genome-wide methylation profiling was conducted with Illumina Infinum HumanMethylation450 BeadChip (Illumina Inc., San Diego, CA), from cord blood plasma aliquots stored at -80°C with methylation levels expressed as beta values ranging from 0 (no methylation) to 1 (complete methylation).

DNA methylation of NFBC1966 at 31 years and NFBC1986 at 16 years were assayed using Infinium HumanMethylation450 BeadChip (Illumina, Inc., San Diego, CA, USA) after bisulphite conversion of genomic DNA performed using the EZ DNA methylation kit according to manufacturer's instructions (Zymo Research, Orange, CA). DNA methylation was measured for 816 randomly selected individuals in NFBC1966 and for 546 randomly selected individuals in NFBC1986. From the NFBC1966 samples, 9 technical replicates, 67 samples that did not reach a call rate of >95% with a detection P-value filter of 10^{-16} and 8 samples with sex mismatch were excluded. From the NFBC1986 samples, 24 technical replicates, 18 samples that did not reach a call rate of >95% applying a detection P-value filter of 10^{-16} and 7 samples with sex inconsistency were excluded. DNA methylation data of both cohorts were normalised using functional normalisation [1] and potential confounding effects of blood cell subtypes were estimated by the Houseman method [2]. After exclusions due to missing covariate info, DNA methylation of 722 NFBC1966 samples and 705 NFBC1986 samples were used in the analyses. The participant's age at methylation measurement was recorded on questionnaires. Maternal age, parity, maternal education and maternal smoking during pregnancy was obtained from the questionnaires administered to the mothers during pregnancy. Maternal smoking was categorised as: not smoked during pregnancy (reference), smoked, but not after second month of pregnancy and smoked after second month of pregnancy. Maternal socioeconomic status was categorised into four groups: professionals, skilled workers, unskilled workers and housewives, farmers and farmer's wives.

Supplemental table 7. Cohort specific methodology regarding EWAS and covariates.

Cohort	Normalisation method	Pre-processing	Quality control	Batch adjustment	Sex	Age	Maternal smoking	Socioeconomic status	Birth weight in grams
HeSVA	Functional normalization	minfi	minfi	ComBat	2 categories: men and women	Continuous	2 categories: nonsmoking in pregnancy, smoking during pregnancy	3 categories based on parental education: primary, secondary and tertiary education	Continuous
PREDO	Functional normalization	minfi	minfi	ComBat	2 categories: men and women	Based on cord blood	3 categories: no smoking during pregnancy, quit in early pregnancy, continued throughout pregnancy	4 categories based on parental education: <high school, high school, college, college for 4+ years	Continuous
GLAKU	Functional normalization	minfi	minfi	ComBat	2 categories: men and women	Continuous	3 categories: no smoking during pregnancy, smoking during pregnancy, smoking at sampling age	3 categories based on parental education: primary, secondary and tertiary education	Continuous
NFBC1986	Functional normalization	minfi	minfi	Functional normalization	2 categories: men and women	Continuous	3 categories: no smoking during pregnancy, quit in early pregnancy, continued throughout pregnancy	4 categories: professionals, skilled workers, unskilled workers and housewives, farmers and farmer's wives	Continuous
NFBC1966	Functional normalization	minfi	minfi	Functional normalization	2 categories: men and women	Continuous	3 categories: no smoking during pregnancy, quit in early pregnancy, continued throughout pregnancy	4 categories: professionals, skilled workers, unskilled workers and housewives, farmers and farmer's wives	Continuous

1 Pidsley, R. et al. A Data-Driven Approach to Preprocessing Illumina 450k Methylation Array Data. *BMC Genomics* **14**, 293 (2013).
2 Houseman, E. A. et al. DNA Methylation Arrays as Surrogate Measures of Cell Mixture Distribution. *BMC Bioinformatics* **13**, 86 (2012).
